# Safety and efficacy of Shatavari in women with low libido: a randomized, double-blind, placebo-controlled trial

**DOI:** 10.25122/jml-2026-0041

**Published:** 2026-07

**Authors:** Madhuchander Satla, Mounika Mudhigonda, Gurunath Surampalli

**Affiliations:** 1 Department of Obstetrics and Gynaecology, Jeevak Hospital – Multispecialty, Hanamkonda, Telangana, India; 2 Department of Obstetrics and Gynaecology, Sairam Multispecialty Hospital, Hanamkonda, Telangana, India; 3 Biofact Research Pvt. Ltd., Visakhapatnam, Andhra Pradesh, India

**Keywords:** Shatavari, *Asparagus racemosus*, sexual wellbeing, low sexual desire, fatigue, sleep disturbance, anxiety, women's health

## Abstract

Women with low libido commonly report reduced desire and diminished arousal, along with negative feelings about their physical and emotional satisfaction and overall wellbeing. In line with this evidence, the present study evaluated the potential of Shatavari root extract (300 mg daily) to support healthy sexual function in women experiencing lifestyle-related reductions in sexual desire. A prospective, randomized, single-center, double-blind, placebo-controlled clinical trial was performed in India. A total of 70 healthy women with low libido characteristics (aged 18–45 years) were allocated to receive active (*n* = 35) or placebo (*n* = 35) for 8 weeks. The primary outcome was the change in total Female Sexual Function Index (FSFI) score; secondary outcomes were validated sexual-function and psychosocial scores and reproductive/stress hormones from baseline to Day 56. Total FSFI improved significantly more with active than placebo (Active +67.96% vs placebo +7.58%; *P* < 0.001), with a between-group mean difference at Day 56 of 3.3 points (95% CI 1.6 to 5.0; *P* = 0.001) and the largest gain in the lubrication domain (*P* < 0.001). The Female Sexual Encounter Profile–Revised increased (+61.9% vs +4.44%; *P* = 0.003); the Hospital Anxiety and Depression Scale decreased (−24.81% vs +7.08%; *P* < 0.001); Patient-Reported Outcomes Measurement Information System Sleep Disturbance Short Form 8b scores improved (−21.83% vs −0.06%); and the Fatigue Severity Scale improved (−60.05% vs −4.45%; *P* < 0.001). Shatavari was well tolerated, with no serious adverse events.

## INTRODUCTION

Sexual function profoundly impacts self-esteem, intimate relationships, and quality of life beyond its fundamental role in reproduction. Yet, despite its considerable importance, female sexual function remains less understood and notably more complex than its male counterpart. Female sexual function is an important part of women’s health and is affected by multiple factors such as psychosexual, contextual, and biological influences. Globally, female sexual vitality represents a prevalent yet frequently under-recognized and studied health challenge, affecting an estimated 30% to 60% of women with some form of dysfunction during their lifetime, highlighting its significant public health burden [1,2]. Studies from various countries highlight low desire conditions, with research showing that 36% of Australian women and 31.5% of Iranian women aged 20–60 years developed new-onset sexual complaints within a 12-month period [3,4]. This pervasive frequency indicates that low libido is not limited to a particular group but represents a widespread issue that can emerge at any stage of adulthood [5]. Meta-analyses further reveal that approximately 41% of reproductive-aged women experience some form of reduced sexual desire, ranking it among the most common health concerns in this demographic [6]. Sexual desire and function are essential components of a woman’s overall health and quality of life that may be compromised by sexual vitality. In women aged 18 to 40, low sexual desire is an increasingly reported concern, yet it remains under-recognized and under-treated due to complex non-hormonal factors.

Reduced sexual desire and function manifest in diverse forms, with arousal disorder the most frequent — affecting nearly half of women with these concerns — followed by reduced lubrication, sexual pain, decreased desire, and difficulty achieving orgasm [1]. Their effects extend beyond physical discomfort, often triggering emotional distress, loss of self-esteem, and relational strain that collectively diminish overall wellbeing [2].

The factors driving women’s sexual wellness vary across life stages and are influenced by hormonal, neuroendocrine, and psychosocial factors [7]. A meta-analysis (21 eligible studies) estimated the pooled prevalence of variations in sexual function at 50.75% among reproductive-age women, which is largely influenced by non-hormonal factors rather than hormonal factors such as chronic psychosocial stress, emotional burnout, lifestyle fatigue, anxiety, and depression [3,8,9]. These factors dysregulate the hypothalamic-pituitary-adrenal (HPA) axis and disrupt critical neurotransmitter networks, including dopaminergic and serotonergic pathways that govern sexual motivation, arousal, and pleasure. Chronic stress and mood disturbances reduce neural activity in the limbic and prefrontal regions—key areas governing emotional regulation and sexual motivation—illustrating how psychological distress can translate into impaired sexual response [1,10].

Unlike women at menopause — in whom reduced sexual wellness is primarily attributed to hormonal decline — lower desire in younger, reproductive-age women is more often associated with chronic psychosocial stress, lifestyle fatigue, poor sleep, and emotional burnout. Menopausal women, by contrast, experience a hormone-driven reduction in sexual vitality characterized by hypoestrogenism, genitourinary atrophy, reduced vascularity, decreased lubrication, and lowered genital sensitivity [11]. Up to 84% of menopausal women report symptoms of the genitourinary syndrome of menopause (GSM), predominantly vaginal dryness and dyspareunia, attributable to estrogen deprivation rather than the psychogenic causes that more often underlie female sexual dysfunction in younger women [7]. Understanding these distinct mechanisms matters, because treatments focused solely on hormone replacement are often less effective in younger women facing predominantly psychosocial and neuroendocrine contributors [7].

Non-pharmacologic interventions such as cognitive behavioral therapy (CBT), which target cognitive and emotional aspects, show limited effectiveness for physiological symptoms such as sexual pain or lubrication difficulties [12]. These findings highlight the current gap between symptomatic pharmacological relief and the multi-factorial physiological and psychological drivers underlying female sexual wellness in reproductive-age women [9,10,13,14].

Shatavari (*Asparagus racemosus*) stands out as a candidate with both empirical validation and mechanistic plausibility. The plant is known as a ‘vajikaran rasayana’ (aphrodisiac), an Ayurvedic category of agents that support sexual performance and wellbeing [14,15]. Preclinical studies show that Shatavari may reduce stress-induced cortisol elevations and oxidative stress in the hippocampus and striatum — brain regions involved in stress regulation and reward processing that may influence sexual function [16].

The traditional use of Shatavari suggests potential benefits for women’s hormonal balance and physiological well-being. Stress-induced oxidation has been proposed to adversely affect female reproductive health, and Shatavari, through its antioxidant properties and bioactive compounds, may mitigate these effects by supporting hormonal balance and reproductive function [5]. The primary bioactive components of *A. racemosus* roots are steroidal saponins, which substantially contribute to its biological activity [11,16]; the plant also contains alkaloids, quercetin, and quercetin glycosides, which further augment its therapeutic properties [11]. *A. racemosus* has traditionally been used to manage conditions of the female reproductive system and to support the immune and nervous systems [16, 17].

The phytoestrogenic compounds in Shatavari, attributed primarily to its steroidal saponins, exhibit estrogen-like activity, making it useful for hormonal modulation [11, 17]. It has been shown to stimulate and regulate the menstrual cycle, support hormonal balance, and support revitalization of the female reproductive system [18,19]. A preclinical study in ovariectomized rats found that ethanolic *A. racemosus* root extract up-regulated brain-derived neurotrophic factor (BDNF) and estrogen receptors (ERα and ERβ) in the cortex and hippocampus, suggesting potential benefits for mood and cognitive functions linked to sexual health [6]. Further mechanistic work using in vitro and in silico docking of Shatavari’s phytochemicals onto estrogen receptor-α revealed binding affinity consistent with phytoestrogenic, estrogen-like activity, supporting modulation of female reproductive physiology [20].

While *A. racemosus* has a long history of use in Ayurvedic medicine for women’s reproductive and hormonal health, most clinical research to date has focused on postmenopausal or hormone-compromised populations, emphasizing effects on the hypothalamic–pituitary–ovarian (HPO) axis and estrogenic activity. However, emerging preclinical studies increasingly suggest that Shatavari may also benefit sexual behavior, stress adaptation, and reproductive resilience in younger populations [6].

No adequately powered trial has evaluated Shatavari specifically in reproductive-age women with low sexual desire, representing a critical evidence gap. To address this, we conducted a trial in women with self-reported low libido to evaluate the effects of *A. racemosus* on these parameters.

## MATERIAL AND METHODS

### Study design and setting

This randomized, double-blind, placebo-controlled clinical trial enrolled healthy women with self-reported low or reduced sexual desire for at least 3 months at one clinical site in India.

The trial aimed to recruit approximately 70 participants, of whom 67 healthy low-libido women completed the 56-day intervention period. Participants were allocated in a 1:1 ratio using computer-generated randomization sequences to one of two groups: (1) Shatavari root extract 300 mg capsules, administered once daily (*n* = 34; total daily dose 300 mg), or (2) matching placebo capsules 300 mg, once daily (*n* = 33; microcrystalline cellulose vehicle). An independent statistician, who was not involved in participant recruitment, enrolment, intervention administration, outcome assessment, or data analysis, generated the random allocation sequence before participant recruitment began using a computer-based random number generator with a 1:1 allocation ratio between the study groups. Allocation concealment was ensured through sequentially numbered, opaque, sealed envelopes prepared by the statistician and kept in a secure location. All envelopes were identical in appearance and were opened strictly in numerical order only after participant eligibility had been confirmed and baseline assessments had been completed. Investigators at the study site enrolled eligible participants and assigned them to treatment groups by opening the next envelope in sequence. The investigators responsible for enrolment had no access to the randomization list and were unaware of future treatment assignments throughout the recruitment process. This procedure ensured allocation concealment and minimized selection bias. Eligible participants were allocated at Visit 2 (baseline, Day 1), and blinded study medication was dispensed based on pre-generated randomization codes held by an independent biostatistician.

**Figure 1:**
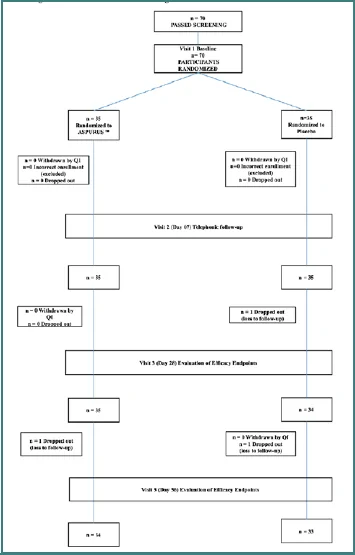
CONSORT 2025 Flow Diagram

Double-blinding was maintained through identical packaging, labeling, capsule appearance, and dosing instructions. Emergency unblinding procedures were available but were not required at any point during the trial. No major protocol modifications occurred after initiation; minor administrative clarifications were reviewed and approved by the IEC before implementation. The study enrolled otherwise healthy women with self-reported low sexual desire. Many participants also reported common lifestyle-related factors, including mild perceived stress, occasional fatigue, or sleep disturbances, which they felt negatively affected their sexual desire and vitality. Women with severe medical, psychiatric, endocrine, or gynecological conditions affecting sexual function are excluded. Full inclusion and exclusion criteria are provided in Table 1, and the participant flow is shown in Figure 1.

**Table 1:** Eligibility criteria for study population

No.	Inclusion criteria	Exclusion criteria
**1**	Sexually active women aged 18–45 years	Use of hormonal contraceptives (intrauterine devices, implants, oral contraceptive pills)
**2**	Stable relationship with a partner for ≥6 months	Pregnant or lactating
**3**	Regular menstrual cycles (24–35 days) for ≥3 months	Use of antidepressant or anxiolytic medication (e.g., SSRIs)
**4**	Vaginal intercourse ≥2–4 times/month for ≥3 months	Untreated or unstable polycystic ovary syndrome, insulin-sensitivity issues, or thyroid disorders
**5**	Body-mass index 18.5–29.9	Undiagnosed pelvic-floor or major endocrine disorders
**6**	Self-reported low or reduced sexual desire on the Decreased Sexual Desire Screener (DSDS)	Recreational drug or nicotine use
**7**	Reduced total Female Sexual Function Index (FSFI) score at screening, consistent with reduced sexual function	Alcohol intake >7 drinks/week
**8**	Reliable, honest, and compliant in the investigator’s judgment	Clinically diagnosed moderate-to-severe psychiatric disorder
**9**	Able to communicate about sexual functioning	History of cancer treatment
**10**	No plan to start new supplements or interventions during the study	Sexually transmitted infection
**11**	Willing to give written informed consent	Untreated hyperprolactinaemia
**12**	Willing to comply with study procedures	Use of herbal extracts within the past 3 months
**13**		Hormone replacement therapy for >3 months
**14**		Active medical, surgical, or gynecological problems
**15**		History of alcohol or tobacco dependence, or substance abuse
**16**		Clinically relevant systemic disease
**17**		Mental condition affecting understanding of the study
**18**		Poor compliance or uncooperative attitude
**19**		Inability to attend follow-up visits
**20**		Hypersensitivity to Shatavari or the study formulation
**21**		Participation in another clinical trial within 3 months
**22**		Any condition deemed unsafe by the investigator

### Decreased Sexual Desire Screener (DSDS)

The DSDS is a validated instrument for the presence and context of low sexual desire in pre-, peri-, and postmenopausal women. It assesses sexual desire specifically and does not screen for or exclude other female sexual disorders (e.g., female sexual arousal disorder, female orgasmic disorder, or sexual pain), although these often co-occur with reduced sexual interest. Consistent with this, the study evaluated supplementation in otherwise healthy women with self-perceived mild-to-moderate low sexual desire and associated concerns. A multi-point item was used to identify common rule-out factors, supporting selection of a population without a primary medical or psychiatric cause for their self-reported experience. The DSDS was scored using a predefined rule-based algorithm on item-level responses [21].

### Investigational products and comparators

#### Active treatment (Shatavari)

The root extract of Shatavari (*Asparagus racemosus* Willd.) was formulated in vegetarian capsules, each containing 300 mg of extract standardized to 5% total Shatavarins. Comprehensive phytochemical analysis was performed to ensure consistency, including quantification of Shatavarins and verification of the absence of microbial contaminants and heavy metal residues [3]. Participants randomized to the active treatment group received one shatavari active capsule daily (300 mg total daily dose of standardized extract). The investigational product was supplied in labeled containers containing the 56-day supply. Both the investigational product (shatavari root extract, ASPURUS) and placebo comparator were manufactured by Waleria Healthtech Pvt. Ltd. under stringent Good Manufacturing Practice (GMP) conditions with documented quality assurance protocols.

#### Placebo comparator

A matching placebo capsule was formulated with microcrystalline cellulose (MCC) 300 mg as the inert vehicle, containing all excipients identical to the active product except the botanical extract. It matched the active capsule in appearance, color, size, texture, odor, and taste to ensure complete blinding of participants and personnel. Packaging, preparation, administration schedule, dose (one capsule daily), method of use, and storage were identical between groups; capsule numbering and labeling were identical, with randomization codes to prevent unblinding.

### Study procedures and visit schedule

The study included five scheduled visits across a 56-day evaluation period. The visit schedule was structured to assess the onset of symptomatic benefit, monitor its trajectory over time, and evaluate the durability of response through successive assessment intervals.

#### Visit schedule and procedures

*Visit 1 (screening and recruitment; up to Day 0)*. During the 15-day screening and 30-day recruitment period, demographic data, medical and medication histories, and current illness details were documented. Informed consent was obtained, followed by recording of vital parameters and a complete clinical examination. Baseline DSDS and Female Sexual Function Index (FSFI) assessments characterized self-reported low sexual desire and established pre-supplementation status.

*Visit 2 (Day 1 — enrolment and randomization)*. Demographic, medical, and current-illness status were confirmed and informed consent verified. Vital parameters (temperature, blood pressure, pulse, respiratory rate) and a complete examination were recorded. Baseline laboratory tests included estradiol, prolactin, free testosterone, dehydroepiandrosterone sulfate (DHEA-S), and cortisol. Mild psychosocial stress, anxiety, fatigue, and sleep disturbance were recorded using the Hospital Anxiety and Depression Scale (HADS), the Fatigue Severity Scale (FSS), and the PROMIS Sleep Disturbance Short Form 8b (PROMIS SD SF-8b), respectively. Sexual function was documented with the FSFI, and vaginal moisture with the modified Schirmer test. Eligible participants were then allocated and enrolled.

*Visit 3 (Day 7 — telephone)*. Early changes were assessed; mean changes were recorded for the FSS and HADS, giving an initial indication of symptom-burden trajectories in the first week.

*Visit 4 (Day 28)*. Demographic, medical, and current-illness status were reviewed and consent reconfirmed. Vital parameters and a complete examination were performed. Assessments included mean changes in the FSFI, FSS, PROMIS SD SF-8b, HADS, and Female Sexual Encounter Profile–Revised (FSEP-R), and vaginal moisture. The Daily Diary of Sexual Events (DDSE) documented real-time activity. Adverse events (AEs) were reviewed.

*Visit 5 (Day 56)*. Status was updated, and consent reconfirmed. Vital signs and a comprehensive examination were performed. Laboratory tests determined mean changes in estradiol, prolactin, free testosterone, DHEA-S, and cortisol. Effects were assessed via mean changes in FSFI, FSS, PROMIS SD SF-8b, HADS, DSDS, and FSEP-R, with vaginal-moisture evaluation. Patient-experienced outcomes used the DDSE and the Low Libido/Low Sex Drive Symptoms Treatment Satisfaction Questionnaire (LLS/LSDS-TSQ). AEs were reviewed and documented.

#### Assessment instruments and outcome measures

The study utilized a robust, multidimensional set of validated psychometric tools to thoroughly assess the multifactorial nature of low libido, capturing sexual, psychological, and functional domains relevant to female sexual health. Low libido is influenced by sexual function, encounter quality, fatigue, and psychosocial wellbeing.

#### Primary outcome measure

*Female Sexual Function Index (FSFI-19)*. The FSFI-19 was selected as the primary outcome because it is the most widely used and psychometrically validated instrument for the assessment of female sexual function. This questionnaire comprised 19 questions regarding sexual function in the last 4 weeks. Questions included: items 1–2 related to sexual desire; items 3–6 to sexual arousal; items 7–10 to sexual lubrication; items 11–13 to orgasm; items 14–16 to sexual satisfaction; and items 17–19 to pain assessment. The reliability of the scale and subscales was determined by calculating Cronbach’s alpha coefficient, with values ≥0.70 indicating good reliability. Mohammadi *et al*. confirmed that the Persian version of the FSFI is valid and reliable. Reliability of the Persian version of the questionnaire for six subscales was calculated by internal consistency using Cronbach’s alpha. The correlation between questions in all subscales was >0.61, which was considered acceptable. The internal consistency of the whole scale was >0.85, which reflected good reliability. Furthermore, based on sensitivity and specificity analysis, Mohammadi *et al*. identified a score of 28 as a desirable cutoff for the identification of women experiencing sexual function concerns [11,17].

The FSFI is a 19-item measure of female sexual function consisting of six domains: desire, arousal, lubrication, orgasm, satisfaction, and pain, allowing a multidimensional evaluation of changes in sexual function relevant to low libido. Scores for the arousal, lubrication, orgasm, and pain domains range from 0 to 6 using Likert-type scales. Desire scores range from 1.2 to 6.0, and satisfaction scores range from 0.8 to 6.0. The total score is the sum of the domain scores and ranges from 2 to 36, and the recall period is the past 4 weeks. Higher scores represent better sexual function [18].

#### Secondary outcome measures

*Daily Diary of Sexual Events (DDSE)*. A regular note of daily sexual events, including subdomains like sexual activity attempt, penetration attempt, orgasm, satisfaction, lubrication, and pain/discomfort, was recorded, providing in-depth information on the intervention’s role in supporting healthy sexual function.

*Female Sexual Encounter Profile-Revised (FSEP-R)*. The FSEP-R is a 10-item instrument intended to assess sexual encounters, including initiation, level of desire, satisfaction with arousal, lubrication, arousal, ability to achieve orgasm, and satisfaction with the sexual encounter. Participants completed the FSEP-R within 24 hours of a sexual encounter. A “sexual encounter” was defined as any act involving sexual contact with genitalia and/or oral mucosa, and includes intercourse, oral sex, and masturbation by self or a partner. Q10 reads “Did you consider this sexual encounter satisfactory for you?” and answers were yes or no. The Female Sexual Encounter Profile-Revised (FSEP-R) is a five-item, event-based measure assessing desire, arousal, genital sensation/lubrication, orgasm, and overall satisfaction after each sexual encounter. Each item is scored dichotomously (Yes = 1, No = 0), and total scores range from 0–5, with higher scores indicating more successful and satisfying sexual responses. Scores of 0–1 reflect minimal response, 2–3 indicate partial response, and 4–5 denote a strong, satisfactory encounter. In clinical research, FSEP-R scores are summarized across encounters to assess an intervention’s effects on sexual encounter quality [19].

*Fatigue Severity Scale (FSS)*. The Fatigue Severity Scale (FSS) was administered to quantify occasional fatigue, a recognized contributor to reduced sexual interest and impaired sexual function in chronic conditions. Previous work has demonstrated significant negative correlations between FSFI scores and FSS scores, indicating that higher fatigue is associated with lower sexual function. The FSS questionnaire is composed of 9 items related to the severity of symptoms commonly present in these patients. Each item is a statement about symptom presence over the past week and is rated on a 7-point Likert scale, from 1 (strong disagreement) to 7 (strong agreement). The total score is the mean of the 9 items, yielding a score range between 1 and 7; a higher mean score indicates greater severity of fatigue symptoms. According to the classification proposed by Johansson *et al.*, participants were categorized into three groups: non-fatigue (FSS ≤ 4.0), borderline fatigue (4.0 < FSS < 5.0), and fatigue (FSS ≥ 5.0) [12].

*Hospital Anxiety and Depression Scale (HADS)*. HADS is a validated instrument for assessing anxiety and depressive symptoms across a broad spectrum of severity. This scale has also been shown to be a valid measure of the severity of these mood disorders; therefore, repeated administration at subsequent clinic visits provides the physician with useful information about progress [7]. In the present study, HADS was employed not to identify clinical mood disorders but to quantify mild psychosocial symptom burden, specifically occasional anxiety, low mood, and general stress, as contributing lifestyle factors to self-reported low sexual desire.

HADS is a 14-item self-report instrument. It includes seven anxiety items (e.g., “I feel tense or wound up,” “Worrying thoughts go through my mind,” “I get a sort of frightened feeling as if something awful is about to happen”) and 7 depression items (e.g., “I still enjoy the things I used to enjoy,” “I feel cheerful,” “I look forward with enjoyment to things”). Each item is scored from 0 to 3 based on symptoms over the past week, with higher scores indicating greater anxiety or depressive severity [7,8].

*PROMIS SD SF-8b*. The PROMIS SD SF-8b was administered to assess sleep disturbance, a symptom known to influence overall well-being, mood, fatigue, and sexual health. The PROMIS SD SF-8b evaluates perceived sleep disturbance over the preceding 7 days and captures multiple dimensions of sleep, including restless sleep, satisfaction with sleep, refreshing sleep, difficulty falling asleep, difficulty staying asleep, problems getting to sleep, adequacy of sleep duration, and overall sleep quality. The scale comprises eight items drawn from the PROMIS sleep disturbance item bank, with item responses summed to generate a total raw score ranging from 8 to 40, where higher scores indicate greater sleep disturbance. In accordance with scoring guidelines, total scores were calculated only when all items were completed; questionnaires with missing item responses were treated as missing. Raw scores were subsequently converted to standardized T-scores to facilitate clinical interpretation. T-scores were categorized as none to slight sleep disturbance (<55), mild sleep disturbance (55.0–59.9), moderate sleep disturbance (60.0–69.9), and severe sleep disturbance (≥70). Including the PROMIS SD SF-8b provided a standardized, validated, and interpretable assessment of sleep-related symptom burden and supported evaluation of whether improvements in sleep disturbance contributed to broader supplementation-related benefits in symptom severity and quality of life [9,13].

### Laboratory and biomarker assessments

Biomarker evaluations were performed at two predefined intervals: Visit 1 (Days 0–30) and end of treatment (Visit 5, Day 56) to characterize supplementation-associated modulation of hormonal and metabolic profiles.

#### Hormonal biomarkers and safety laboratory parameters

Reproductive and stress-axis hormonal profiling was conducted using high-sensitivity, analytically validated immunoassay platforms to ensure optimal accuracy and detection fidelity across physiological concentration ranges. The endocrine panel included estradiol (pg/mL), prolactin, morning serum cortisol, dehydroepiandrosterone sulfate (DHEA-S), and free testosterone, each quantified using rigorously calibrated assay systems referenced to established clinical standards. These biomarkers were selected based on their established roles as endocrine determinants of female sexual desire and function, and to characterize potential hormonal mediators of intervention response. Pre-analytical procedures, particularly for cortisol and free testosterone, were standardized to minimize diurnal, metabolic, and procedural variability [14]. In addition, hepatic safety surveillance was undertaken through a comprehensive liver function test (LFT) panel, encompassing aspartate aminotransferase (AST), alanine aminotransferase (ALT), alkaline phosphatase (ALP), total bilirubin, and direct bilirubin. All biochemical analyses were performed at centralized, accredited laboratories utilizing harmonized operating procedures, stringent internal quality assurance controls, and cross-platform calibration, thereby supporting methodological robustness, inter-assay reproducibility, and high scientific reliability across all study time points [15].

#### Vaginal moisture assessment (modified Schirmer test)

Vaginal dryness was objectively quantified using the modified Schirmer test, a physiological measure adapted from the classic Schirmer tear test originally developed to assess lacrimal secretion. Recent research supports this adaptation for gynecological use, showing that calibrated filter paper strips placed against the lateral vaginal wall for a defined period (e.g., 5 minutes) accurately capture vaginal fluid levels via capillary action and wetting distance in millimeters, providing a direct, continuous indicator of mucosal moisture. In a prospective cohort study, this modified approach distinguished women with genitourinary syndrome of menopause from asymptomatic controls, with significantly lower wetting measurements observed in symptomatic subjects and strong correlations with established clinical indices including vaginal pH, Vaginal Health Index (VHI), and patient-reported dryness scores. These findings establish the modified Schirmer test as a reproducible, minimally invasive objective tool that complements subjective symptom scales and may be particularly useful for longitudinal assessment of interventions targeting vaginal atrophy and dryness. In addition, related research has explored the use of Schirmer tear test strips to evaluate physiological vaginal lubrication in response to sexual arousal stimuli, highlighting broader applications of capillary-based moisture measurements in genital mucosal research [22]. Feasibility evidence supports extending this approach to younger populations: Schirmer test strips showed sensitivity to increases in physiological lubrication in response to sexual arousal stimuli, with moderate correlations to self-reported genital arousal (r = 0.41) and lubrication (r = 0.30). This supports the test’s mechanistic rationale that vaginal moisture is a cardinal component of sexual arousal and may mediate improvements in comfort, pleasure, and satisfaction. The modified Schirmer test thus provides an objective genital measure that complements subjective FSFI lubrication items, strengthening inferences about the arousal–satisfaction pathway. While validation data predominate in postmenopausal populations, demonstrated sensitivity to arousal-induced changes provides a defensible, exploratory rationale for use in the reproductive-age group.

### Outcome definitions

#### Primary outcome

##### Primary study outcome

The primary endpoint was the mean change in total FSFI score, administered at screening (Visit 1), baseline/enrolment (Visit 2, Day 1), week 4 (Visit 3), and week 8 (Visit 4, end of study). The study assessed changes in both total and domain-specific FSFI scores as the principal measure of improvement in low libido [11]. FSFI total score ranged from 2 to 36, with higher values indicating better sexual function, so that the direction of clinically meaningful change is unambiguous. The primary analysis was conducted in the intention-to-treat (ITT) population, defined as all randomized participants who provided at least one post-baseline FSFI assessment. All randomized participants were included in the analysis according to their assigned treatment group, and missing data were handled as specified in the statistical analysis plan.

##### Secondary and safety outcomes

Secondary and exploratory study endpoints encompassed quantitative evaluation of vaginal moisture using validated objective methodologies, including the modified Schirmer test [4].

Psychometric assessments were conducted via the HADS, PROMIS SD SF-8b, and the FSS scales. Event-specific sexual response dynamics encompassing arousal and post-encounter satisfaction were evaluated using the FSEP-R, administered within 24 hours of each sexual event. Endocrine profiling, including serum estradiol, prolactin, free testosterone, DHEA-S, and cortisol, was performed at baseline (Day 1) and day 56 to characterize supplementation-associated hormonal modulation.

### Statistical methods and sample size

The primary endpoint was analyzed using a two-way repeated-measures ANOVA with treatment group as the between-subject factor and study day as the within-subject factor. The interaction between treatment and time was evaluated. When significant overall effects were detected, post hoc multiple-comparison tests (Tukey’s or Bonferroni’s, as appropriate for the analysis) were performed to identify between-group differences. Baseline (Day 0) measurements were examined to confirm comparability between groups; therefore, no baseline adjustment was applied. Data are presented as mean ± SD. Secondary continuous outcomes were analyzed using appropriate parametric or non-parametric methods depending on data distribution and underlying assumptions. Categorical variables were summarized using frequencies and percentages and compared using the Chi-square test or Fisher’s exact test, as appropriate. All statistical tests were two-sided, and a *P* value < 0.05 was considered statistically significant. Continuous variables were summarized using descriptive statistics (*n*, mean, median, SD, coefficient of variation, and range) by treatment group and overall. Analyses were conducted using SPSS Software Version 30. Within-group comparisons applied parametric tests for normally distributed variables and the Wilcoxon signed-rank test for non-normal data. Repeated-measures ANOVA was used to evaluate longitudinal changes from baseline, while between-group comparisons utilized unpaired *t*-tests or Mann–Whitney U tests based on distributional characteristics. Repeated-measures ANOVA revealed a significant treatment × time interaction, indicating differential trajectories of symptom change between groups across the 6-week period, followed by post-hoc tests at individual time points.

The study targeted 70 participants, anticipating a ~10% dropout rate, with an expected final sample of 60 healthy, sexually active women aged 18–45 years in stable relationships, with regular menstrual cycles (24–35 days), body-mass index 18.5–29.9, and vaginal intercourse ≥2–4 times/month, who self-reported moderate fatigue, sleep disturbance, or stress affecting desire or energy and screened positive for low libido on the DSDS (Table 2). Sample size was calculated using G*Power 3.1 assuming: (1) primary outcome of FSFI-19 total score change from baseline to week 6, (2) medium effect size (d = 0.6), (3) alpha = 0.05 (two-tailed), power = 0.80, and (4) dropout rate of 10%. This yielded 30 participants per arm (total *N* = 60), with recruitment of 70 to account for potential attrition. The trial was powered for the week-6 endpoint, while Day-56 analyses were regarded as key secondary or confirmatory endpoints; post-hoc power or sensitivity considerations for Day 56 were added, wherever necessary, together with a brief justification that the original sample size remained sufficient to detect clinically relevant differences at Day 56, given the expected effect size and observed variability.

**Table 2:** Participant demographics comparing active and placebo groups

	Active, *n* =35	Placebo, *n* = 35	*P* values
Age years, mean ± SD	31.69 ± 6.39	32.74 ± 7.35	Ns
Weight (kg), mean ± SD	66.12 ± 7.67	65.14 ± 9.31	Ns
BMI (kg/m2), mean ± SD	24.1 ± 3.0	24.3 ± 3.1	Ns

## RESULTS

### Participant baseline characteristics

Among the 70 randomized participants, three did not complete the study (e.g., withdrew consent, were lost to follow-up, or discontinued treatment), resulting in 67 completers. Primary efficacy analyses were performed in the ITT population (*n* = 70), while supportive analyses were also conducted in the completed/evaluable cohort (*n* = 67), with minimal withdrawals, high adherence, and comparable baseline demographic and clinical characteristics between groups. At screening, all participants self-reported low or reduced sexual desire, which was further characterized using the DSDS. The participant flow diagram (Figure 1) summarizes withdrawals and missing data, and sensitivity analyses assessed the impact of withdrawals on the primary endpoint.

### Primary study outcomes

#### FSFI-19

Both treatment groups demonstrated significant within-group improvements in total FSFI scores relative to baseline across all post-baseline evaluations (*P* < 0.001), consistent with the well-recognized placebo responsiveness in studies of female sexual desire and function. Despite this parallel improvement, between-group analyses showed that the active group consistently improved more than placebo at every assessed time point (Table 3). By Day 28, the active group improved by 20.76 ± 4.18 from baseline versus 14.39 ± 3.74 in placebo (between-group *P* < 0.001), the earliest point of measurable divergence, with a further gain by Day 56 (active 24.69 ± 4.19; placebo 14.03 ± 3.32; *P* < 0.001). No significant difference was detected at Day 1, reflecting an expected early latency phase before supplementation-related improvements in sexual wellness became clinically evident. FSFI total score improved by a mean of 4.8 points in group A versus 1.5 points in group B at Day 56, a between-group difference of 3.3 points (95% CI, 1.6 to 5.0; *P* = 0.001), which exceeds proposed thresholds for a clinically meaningful difference in FSFI.

**Table 3:** Comparison of mean changes in sexual function, vaginal moisture, psychological well-being, and fatigue scores between the active and placebo groups from baseline through Day 56 of supplementation

No.	Scales	Before supplementation Mean ± SD Day 01		P*	After supplementation Mean ± SD Day 7		P*	After supplementation Mean ± SD Day 28		P*	After supplementation Mean ± SD Day 56		P*
		Active (*n* = 34)	Placebo (*n* = 33)		Active (*n* = 34)	Placebo (*n* = 33)		Active (*n* = 34)	Placebo (*n* = 33)		Active (*n* = 34)	Placebo (*n* = 33)	
**1**	**FSFI**	14.7 ± 2.59	15.18 ± 4.23	0.572				20.76 ± 4.18	14.39 ± 3.74	<.001	24.69 ± 4.19	14.03 ± 3.32	<.001
**2**	**FSEP-R**	10.34 ± 4.97	11.49 ± 5.29	0.346				14.49 ± 4.81	11.91 ± 4.85	0.03	16.74 ± 5.97	12 ± 4.44	0.001
**3**	**M.S. Test**	8.31 ± 2.92	8.6 ± 3.66	0.734				10.29 ± 3.08	8.41 ± 2.93	0.007	13.53 ± 3.49	8.12 ± 3.19	<.001
**4**	**PROMIS SD SF-8b**	66.46 ± 4.66	66.75 ± 4.85	0.842				55.32 ± 5.09	66.5 ± 4.82	<.001	51.95 ± 4.49	66.79 ± 4.98	<.001
**5**	**HADS**	11.89 ± 3.22	12 ± 3.1	0.81	11.63 ± 2.9	11.97 ± 2.77	0.542	10.8 ± 2.59	12.38 ± 2.09	0.002	8.94 ± 3.03	12.85 ± 2.5	<.001
**6**	**FSS**	38.57 ± 7.5	39.57 ± 6.95	0.565	36.57 ± 7.19	40.46 ± 6.93	0.015	27.06 ± 5.12	40.76 ± 7.34	<.001	15.41 ± 3.07	41.33 ± 7.31	<.001

Values are presented as mean ± SD. Active (*n* = 34) and placebo (*n* = 33) groups were compared using independent-samples t-tests at each time point (P*). Statistical significance was set at *P* < 0.05 (*P* < 0.001, highly significant); ns indicates *P* ≥ 0.05. Higher scores indicate improvement for FSFI, FSEP-R, and M.S. Test, whereas lower scores indicate improvement for HADS and FSS. All scales were validated for menopausal symptom assessment.

#### Desire domain

Baseline desire scores were equivalent across treatment arms (*P* = 0.765). Over successive assessments at Day 28, the active group produced a 33.91% (8.49 ± 2.12) improvement compared with −7.29% (5.85 ± 2) in the placebo group (*P* < 0.001). At the final Day 56 visit, the active group demonstrated a 34.54% (8.53 ± 2) improvement, compared with the 8.24% (5.79 ± 1.9) improvement observed with placebo (*P* < 0.001) (Table 4).

**Table 4:** Between-group comparison of FSFI subdomain scores reflecting sexual desire, arousal, lubrication, orgasm, satisfaction, and pain in women with low libido receiving active treatment or placebo at baseline (Day 1) and after supplementation at Day 28 and Day 56

No.	FSFI	Before supplementation Mean ± SD Day 1		P*	After supplementation Mean ± SD Day 28		P*	After supplementation Mean ± SD Day 56		P*
		Active (*n* = 34)	Placebo (*n* = 33)		Active (*n* = 34)	Placebo (*n* = 33)		Active (*n* = 34)	Placebo (*n* = 33)	
1	**Desire**	6.34 ± 2.04	6.31 ± 2.22	0.765	8.49 ± 2.12	5.85 ± 2	<.001	8.53 ± 2	5.79 ± 1.9	<.001
2	**Arousal**	6.83 ± 1.54	5.89 ± 2.05	0.054	8.89 ± 2.03	5.41 ± 1.76	<.001	11.2 ± 2.18	5.42 ± 1.68	<.001
3	**Lubrication**	5.4 ± 1.48	6 ± 2.1	0.281	8.23 ± 2.31	5.85 ± 1.96	<.001	10.44 ± 2.51	5.52 ± 2.02	<.001
4	**Orgasm**	5.94 ± 1.68	6.77 ± 2.28	0.088	9.06 ± 2.86	6.68 ± 2.06	0.001	11.09 ± 2.54	6.21 ± 1.9	<.001
5	**Satisfaction**	6.66 ± 1.16	6.94 ± 1.63	0.546	9.69 ± 2.52	6.76 ± 1.37	<.001	11.97 ± 2.38	6.67 ± 1.34	<.001
6	**Pain**	5.54 ± 2.29	5.89 ± 2.05	0.453	7.6 ± 2.77	5.44 ± 1.74	0.001	10 ± 2.51	5.33 ± 1.59	<.001

Values are presented as mean ± standard deviation (SD). Active (*n* = 34) and placebo (*n* = 33) groups were compared using independent-samples t-tests at each assessment point (P*). Statistical significance was set at *P* < 0.05, with *P* < 0.001 indicating a highly significant difference; ns denotes *P* ≥ 0.05.

#### Arousal domain

No baseline difference was identified for the arousal domain (*P* = 0.054). By Day 28, the active group improved by 30.16% (8.89 ± 2.03), notably more than the −8.15% (5.41 ± 1.76) change in the placebo group (*P* < 0.001). At Day 56, the improvement continued to favor the active group (63.98%, 11.2 ± 2.18) over placebo (7.98%, 5.42 ± 1.68) (*P* < 0.001) (Table 4).

#### Lubrication domain

Baseline lubrication scores were comparable between groups (*P* = 0.281). At Day 28, lubrication was 8.23 ± 2.31 in the active group versus 5.85 ± 1.96 in the placebo group (*P* < 0.001). This persisted at Day 56, where the active group (93.33%, 10.44 ± 2.51) continued to improve over placebo (8%, 5.52 ± 2.02) (*P* < 0.001), consistent with improved vaginal lubrication supporting comfortable and satisfying sexual activity (Table 4).

#### Orgasm domain

Baseline orgasmic function did not differ significantly between groups (*P* = 0.088). At Day 28, the active group improved by 52.53% (9.06 ± 2.86) versus −1.33% (6.68 ± 2.06) in the placebo group (*P* = 0.001). At Day 56, the difference persisted: active 86.70% (11.09 ± 2.54) versus placebo 8.27% (6.21 ± 1.9) (*P* < 0.001) (Table 4).

#### Satisfaction domain

Baseline satisfaction scores were comparable between the active and placebo groups (*P* = 0.546). At Day 28, active improved by 45.50% versus 2.59% (placebo) (*P* < 0.001); at Day 56, active was 79.73% versus placebo 3.89% (*P* < 0.001) (Table 4).

#### Pain domain

Sexual pain scores at baseline did not differ significantly between groups (*P* = 0.453). By Day 28, the active group demonstrated a 37.18% (7.6 ± 2.77) improvement, whereas the placebo group showed 7.64% (5.44 ± 1.74) improvement (*P* = 0.001). At Day 56, the active group (80.51%, 10 ± 2.51) continued to show better sexual pain scores relative to placebo (9.51%, 5.33 ± 1.59; *P* < 0.001; Table 3).

### Secondary study outcomes

#### Daily Diary of Sexual Events (DDSE)

DDSE outcomes are summarized day-wise as mean ± SD for the active (*n* = 34) and placebo (*n* = 33) groups, with between- and within-group comparisons (Table 5).

**Table 5:** Data of daily diary of sexual events

DAYWISE												
Group	Active vs placebo 1st month		*P*	Active vs placebo 2nd month		*P*	Active 1 vs 2 months		*P*	Placebo 1 vs 2 months		*P*
Questions	Active	Placebo		Active	Placebo		1st month	2nd month		1st month	2nd month	
**Sexual Activity Attempted-%**	54.67 ± 3.73	44.52 ± 0.17	<.001	58.87 ± 6.98	41.07 ± 0.22	<.001	54.67 ± 3.73	58.87 ± 6.98	0.01	44.52 ± 0.17	41.07 ± 0.22	<.001
**Penetration Attempted-%**	37.89 ± 3.01	30.89 ± 0.17	<.001	40.89 ± 5.38	28.97 ± 0.19	<.001	37.89 ± 3.01	40.89 ± 5.38	0.01	30.89 ± 0.17	28.97 ± 0.19	<.001
**Level of desire**	3.21 ± 0.26	2.65 ± 0.16	<.001	3.58 ± 0.39	2.7 ± 0.04	<.001	3.21 ± 0.26	3.58 ± 0.39	<.001	2.65 ± 0.16	2.7 ± 0.04	0.22
**Lubrication**	2.42 ± 0.21	2.01 ± 0.15	<.001	2.71 ± 0.39	1.99 ± 0.04	<.001	2.42 ± 0.21	2.71 ± 0.39	0	2.01 ± 0.15	1.99 ± 0.04	0.5
**Satisfaction**	2.83 ± 0.22	2.55 ± 0.17	<.001	3.27 ± 0.44	2.44 ± 0.06	<.001	2.83 ± 0.22	3.27 ± 0.44	<.001	2.55 ± 0.17	2.44 ± 0.06	0.001
**Orgasm**	0.65 ± 0.09	0.62 ± 0.09	0.34	0.55 ± 0.08	0.65 ± 0.09	<.001	0.65 ± 0.09	0.65 ± 0.09	0.77	0.62 ± 0.09	0.65 ± 0.09	0.38
**Pain/discomfort**	0.26 ± 0.1	0.32 ± 0.09	0.01	0.19 ± 0.12	0.27 ± 0.1	0.01	0.26 ± 0.1	0.19 ± 0.12	0.01	0.32 ± 0.09	0.27 ± 0.1	0.03
**Partner satisfaction**	0.55 ± 0.07	0.52 ± 0.08	0.1	0.62 ± 0.09	0.53 ± 0.09	<.001	0.55 ± 0.07	0.62 ± 0.09	0	0.58 ± 0.1	0.53 ± 0.09	0.07

DDSE data are presented as mean ± SD. Active group (*n* = 34), Placebo (*n* = 33). P* denotes *P* values from independent samples test comparing groups at each individual point.

#### Between-group comparisons (active vs placebo)

*Sexual activity and behavior*. The percentage of days with sexual activity attempted was significantly higher in the active group compared with placebo at both Month 1 and Month 2 (*P* < 0.001), reflecting greater willingness to engage in sexual activity with active supplementation. Similarly, penetration attempts (%) were significantly greater in the active group at both time points (*P* < 0.001).

*Desire, arousal, and satisfaction domains*. The level of sexual desire, lubrication, and overall satisfaction scores were all significantly higher in the active group than in the placebo group at both Month 1 and Month 2 (*P* < 0.001 for all), demonstrating supplementation-related improvements across core sexual function domains.

*Orgasm and partner satisfaction*. No significant between-group difference was observed in orgasm frequency at Month 1 (*P* = 0.34); however, a significant difference emerged at Month 2 (*P* < 0.001), supporting the active group and indicating a delayed but meaningful supplementation effect.

Partner satisfaction scores did not differ significantly between groups at Month 1 (*P* = 0.10) but were significantly higher in the active group by Month 2 (*P* < 0.001).

Pain/discomfort scores for pain or discomfort during sexual activity were significantly lower in the active group compared with placebo at both months (*P* = 0.01), supporting greater physical comfort during sexual activity with intervention.

#### Within-group comparisons (Month 1 vs Month 2)

In the active group, there were significant improvements from Month 1 to Month 2 observed in: sexual activity attempted, penetration attempted, desire, lubrication, partner satisfaction (all *P* ≤ 0.01). Pain/discomfort scores showed a greater reduction over time (*P* = 0.01). Orgasm frequency did not change significantly within the active group (*P* = 0.77).

The placebo group showed no meaningful improvements across most domains. Small statistical changes were observed in certain parameters (e.g., pain/discomfort) but were inconsistent and did not reflect a sustained or clinically relevant trend. Desire, lubrication, satisfaction, and partner satisfaction remained largely unchanged over time.

### Fatigue assessment

#### Fatigue Severity Scale (FSS)

Both groups showed significant change in FSS at Day 7 (*P* = 0.015; active 5.19% [36.57 ± 7.19], placebo −2.25% [40.46 ± 6.93]), Day 28 (*P* < 0.001; active 29.84% [27.06 ± 5.12], placebo −3.01% [40.76 ± 7.34]), and Day 56 (*P* < 0.001; active −60.05% [15.41 ± 3.07], placebo −4.45% [41.33 ± 7.31]) relative to baseline (*P* = 0.565) (Table 2). The progressive reduction in fatigue scores observed in the active group, relative to the minimal placebo response, indicates a clinically relevant benefit for everyday fatigue.

### Anxiety and depression assessment

#### Hospital Anxiety and Depression Scale (HADS)

Baseline HADS total scores did not differ significantly between the active and placebo groups (*P* = 0.81), confirming comparable psychological status at study entry. Reductions in overall HADS were seen at Day 7 (*P* = 0.542; active 2.19% [11.63 ± 2.9], placebo 0.25% [11.97 ± 2.77]), Day 28 (*P* = 0.002; active 9.17% [10.8 ± 2.59], placebo −3.17% [12.38 ± 2.09]), and Day 56 (*P* < 0.001; active 24.81% [8.94 ± 3.03], placebo −7.08% [12.85 ± 2.5]) (Table 2). The extent of psychological improvement was consistently greater in the Shatavari cohort, whereas the placebo group exhibited only minimal change across visits. This pattern indicates a clinically meaningful reduction in everyday psychological burden, reinforcing the multidimensional beneficial effect of Shatavari in women with low sexual desire.

#### Vaginal moisture assessment (modified Schirmer test)

**Figure 2:**
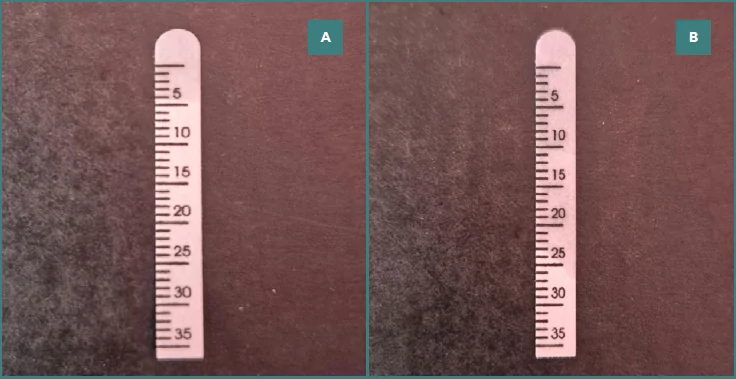
Vaginal moisture measured by the modified Schirmer test. Representative paper strips showing vaginal fluid captured by capillary action, quantified as the wetted length (mm). A, **active group at Day 1, wetted length 4 mm**. B, **active group at Day 56, wetted length 23 mm. Published with participant consent**.

The modified Schirmer test showed comparable baseline vaginal moisture between the active and placebo groups (*P* = 0.734), confirming equivalent genital hydration at study entry. Vaginal wetting length increased progressively with supplementation, favoring the active group at Day 28 (*P* = 0.007; active 23.83% [10.29 ± 3.08] vs placebo −2.21% [8.41 ± 2.93]) and Day 56 (*P* < 0.001; active 62.82% [13.53 ± 3.49] vs placebo −5.58% [8.12 ± 3.19]; 95% CI, 12.31–14.75 for active and 6.99–9.25 for placebo) (Table 2; Figure 2). Between-group analyses consistently favored the active group, whereas placebo responses were minimal or variable. These findings indicate a clinically meaningful improvement in vaginal moisture, supporting a role for Shatavari in improving genital hydration and sexual comfort in women with low sexual desire.

### Quality, satisfaction, and arousal response assessment

#### Female Sexual Encounter Profile-Revised (FSEP-R)

Baseline encounter quality was comparable between groups, with no significant difference. Both groups improved on overall FSEP-R scores at Day 28 (*P* = 0.03; active 40.14% [14.49 ± 4.81], placebo 3.66% [11.91 ± 4.85]) and Day 56 (*P* = 0.001; active 61.90% [16.74 ± 5.97], placebo 4.44% [12 ± 4.44]) relative to baseline (*P* = 0.346) (Table 2). Across visits, the active group showed the greater improvement, a clinically meaningful advantage over placebo.

### Sleep disturbance and sleep quality assessment

#### PROMIS SD SF-8b

Both groups changed significantly in PROMIS SD SF-8b sleep-disturbance scores at Day 28 (*P* < 0.001; active 16.76% [55.32 ± 5.09], placebo 0.37% [66.5 ± 4.82]) and Day 56 (*P* < 0.001; active 21.83% [51.95 ± 4.49], placebo −0.06% [66.79 ± 4.98]; 95% CI 50.41–53.49 for active and 65.08–68.50 for placebo) relative to baseline (*P* = 0.842) (Table 2). The magnitude and progressive nature of sleep disturbance score reductions observed in the active group, relative to the minimal placebo response, indicates a clinically beneficial effect on sleep quality and continuity. Notably, these improvements were large enough to shift participants into lower PROMIS sleep disturbance T-score categories, reflecting a clinically meaningful normalization of sleep patterns.

#### Hormonal parameters

**Figure 3:**
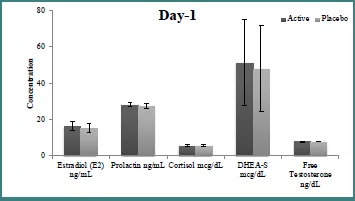
Concentration of serum biomarkers quantified on Day 1 of the study. The data presented here are the mean ± SD of the active group (*n* = 34) and the placebo group (*n* = 33). Statistical significance was calculated using ANOVA, and the significant values were represented as * P ≤ 0.05.

**Figure 4:**
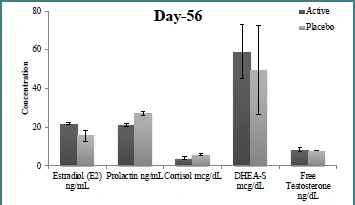
Concentration of serum biomarkers quantified on Day 56 of the study. The data presented here are mean ± SD values of the active group (*n* = 34) and placebo group (*n* = 33). Statistical significance was calculated using ANOVA, and the significant values were represented as * P ≤ 0.05.

Participants receiving active treatment showed modest yet measurable modulation in key endocrine biomarkers, including estradiol (pg/mL), prolactin, cortisol, DHEA-S, and free testosterone, by Day 56, corresponding with the clinical improvements observed (Table 6; Figure 3 and 4).

**Table 6:** Between-group comparison of circulating sex- and stress-hormone concentrations in women with low libido receiving Shatavari or placebo, at baseline (Day 1) and after 56 days of supplementation

No.	Hormones	Before supplementation Mean ± SD Day 1		P*	After supplementation Mean ± SD Day 56		P*
		Active (*n* = 34)	Placebo (*n* = 33)		Active (*n* = 34)	Placebo (*n* = 33)	
**1**	**Estradiol (E2) ng/mL**	16.35 ± 2.35	15.25 ± 2.72	0.073	22.02 ± 0.62	15.57 ± 2.84	<.001
**2**	**Prolactin ng/mL**	28.1 ± 1.18	27.53 ± 1.2	0.058	21.21 ± 0.96	26.98 ± 1.11	<.001
**3**	**Cortisol mcg/dL**	5.82 ± 0.48	5.7 ± 0.6	0.353	3.96 ± 0.76	5.83 ± 0.63	<.001
**4**	**DHEA-S mcg/dL**	51.42 ± 23.47	48.2 ± 23.51	0.569	59.13 ± 14.07	49.47 ± 23.1	0.03
**5**	**Free Testosterone ng/dL**	7.75 ± 0.43	8.02 ± 0.17	0.075	8.53 ± 0.92	8.14 ± 0.03	0.062

Values are presented as mean ± standard deviation (SD). Active (*n* = 34) and placebo (*n* = 33) groups were compared using independent-samples *t*-tests at each time point (P*). Statistical significance was defined as *P* < 0.05, with *P* < 0.001 indicating a highly significant difference; ns denotes *P* ≥ 0.05. Hormonal changes are presented in their respective clinical units.

#### A. Estradiol (pg/mL)

Estradiol levels showed a relative increase at Day 56 versus baseline in the active group (baseline: 16.35 ± 2.35 pg/mL; Day 56: 22.02 ± 0.62 pg/mL; *P* < 0.001), whereas placebo-related changes were minimal (baseline: 15.25 ± 2.72 pg/mL; Day 56: 15.60 ± 2.79 pg/mL). Intergroup comparisons demonstrated a statistically significant difference (*P* < 0.001), suggesting that active supplementation supports estrogenic normalization.

#### B. Prolactin

Prolactin concentrations showed a modulation at Day 56 compared with baseline in the active group (baseline: 28.1 ± 1.18 ng/mL; Day 56: 21.21 ± 0.96 ng/mL; *P* < 0.001), while placebo participants exhibited no meaningful change (baseline: 27.53 ± 1.2 ng/mL; Day 56: 26.98 ± 1.11 ng/mL). Between-group analysis confirmed statistical significance (*P* < 0.001).

#### C. Cortisol

Serum cortisol levels decreased significantly at Day 56 relative to baseline in the active group (baseline: 5.82 ± 0.48 µg/dL; Day 56: 3.91 ± 0.75 µg/dL; *P* < 0.001), whereas reductions in the placebo group were modest or absent (baseline: 4.3 ± 0.68 µg/dL; Day 56: 4.15 ± 0.60 µg/dL). Intergroup comparisons were statistically significant (*P* < 0.001), suggesting that active supplementation reduced stress.

#### D. DHEA-S

DHEA-S levels increased significantly from baseline to Day 56 in the active group (baseline: 51.42 ± 23.47 µg/dL; Day 56: 59.13 ± 14.07 µg/dL; *P* = 0.03), while placebo-associated changes remained negligible (baseline: 48.2 ± 23.51 µg/dL; Day 56: 49.47 ± 23.1 µg/dL). A significant between-group difference was observed (*P* < 0.001).

#### E. Free testosterone

Free testosterone concentrations demonstrated a significant increase at Day 56 compared with baseline in participants receiving active supplementation (baseline: 7.75 ± 0.43 pg/mL; Day 56: 8.53 ± 0.92 pg/mL; *P* < 0.001), whereas placebo effects were minimal (baseline: 8.02 ± 0.17 pg/mL; Day 56: 8.14 ± 0.03 pg/mL). Intergroup analysis confirmed statistical significance (*P* < 0.003), suggesting continuing improvements in sexual desire, arousal, and overall sexual function.

#### Intervention satisfaction

##### Low libido or low sex drive Symptoms Treatment Satisfaction Questionnaire (LLS/LSDS-TSQ)

Treatment satisfaction, assessed using the LLS/LSDS-TSQ, improved more in the active group than in placebo. By the final study visit, mean LLS/LSDS-TSQ scores increased by 33.12 ± 3.52 in the active group versus 21.94 ± 4.02 in the placebo group. These findings suggest superior patient-perceived satisfaction with active treatment in alleviating low sexual desire symptoms.

### Safety outcomes

#### Adverse events

The safety profile of the active intervention was favorable, with no adverse events (AEs) reported in the active arm and no serious adverse events (SAEs) observed in either treatment group throughout the 56-day study period. No participant required medical intervention or hospitalization at any stage. Only two participants reported mild treatment-emergent gastrointestinal symptoms, including nausea, abdominal discomfort, bloating, and occasional loose stools. These events were transient, self-limiting, and resolved spontaneously without specific medical intervention. No participant withdrew from the study because of these symptoms. Overall, the investigational product was well tolerated, and no clinically significant safety concerns were identified throughout the study duration.

#### Laboratory safety parameters

A comprehensive evaluation of laboratory safety parameters at the 8-week endpoint (Day 56) demonstrated that all measures remained within normal clinical limits for both treatment groups, with no clinically meaningful differences observed between them.

#### LFT parameters

All liver function test (LFT) parameters—including total, direct, and indirect bilirubin; serum glutamic-oxaloacetic transaminase (SGOT); serum glutamic-pyruvic transaminase (SGPT); lactate dehydrogenase (LDH); and serum protein, albumin, and globulin concentrations—remained stable across the 56-day intervention period, with no statistically significant differences observed between the active and placebo groups at Day 56 compared with baseline (all *P* > 0.05). Both groups exhibited values consistently within established clinical reference ranges, supporting the hepatic safety of the active intervention, suggesting that the supplementation did not adversely influence metabolic or detoxification pathways. These findings collectively confirm that hepatic function was preserved throughout the study period, supporting the favorable safety profile of the active formulation in women with low sexual desire (Table 7).

**Table 7:** Baseline and post-intervention liver function parameters and vital signs following 56 days of active supplementation compared with placebo

No		Before supplementation Mean ± SD Day 01		P*	After supplementation Mean ± SD Day 56		P*
	Liver Function Test (LFT)	Active (*n* = 35)	Placebo (*n* = 35)		Active (*n* = 34)	Placebo (*n* = 33)	
**1**	Albumin (Serum Albumin)	4.17 ± 0.41	4.34 ± 0.42	0.101	4.18 ± 0.64	4.24 ± 0.58	0.689
**2**	Alkaline Phosphatase (ALP)	78.11 ± 21.59	82.14 ± 20.6	0.343	78.89 ± 23.85	79.95 ± 19.89	0.845
**3**	Alanine Aminotransferase (ALT)	23.26 ± 7.26	25.71 ± 8.29	0.193	23.24 ± 7.41	25.19 ± 8.17	0.31
**4**	Aspartate Aminotransferase (AST)	27.22 ± 7.95	28.08 ± 7.12	0.686	27.14 ± 8.57	28.07 ± 7.68	0.642
**5**	Gamma-Glutamyl Transferase (GGT)	27.45 ± 10.71	28.24 ± 11.62	0.819	26.55 ± 11.29	29.06 ± 11.68	0.378
**6**	Total Bilirubin	0.62 ± 0.31	0.7 ± 0.3	0.311	0.62 ± 0.33	0.67 ± 0.29	0.518
	**Vitals**						
**7**	SBP	113.17 ± 6.05	106.66 ± 9.07	0.001	105.67 ± 8.06	106.21 ± 11.03	0.82
**8**	DBP	75.83 ± 3.94	76.17 ± 4.27	0.728	76.06 ± 9.28	74.53 ± 8.97	0.495
**9**	Body Temp	36.59 ± 0.14	36.6 ± 0.14	0.911	36.62 ± 2.68	36.94 ± 2.7	0.638
**10**	Pulse Rate	72.4 ± 2.06	72.71 ± 2.7	0.699	70.25 ± 6.19	72.32 ± 7.24	0.212
**11**	Respiratory Rate	16.54 ± 1.17	16.69 ± 1.18	0.574	16.97 ± 1.86	16.41 ± 2.02	0.209

Values are presented as mean ± SD, with *P*-values representing between-group comparisons (active vs placebo) at each time point. Liver function parameters included serum albumin, ALP, ALT, AST, GGT, and total bilirubin, while vital signs comprised systolic and diastolic blood pressure, body temperature, pulse rate, and respiratory rate.

#### Vital signs and physical examination

Across the 56-day study period, all vital sign parameters—including systolic blood pressure (SBP), diastolic blood pressure (DBP), body temperature, pulse rate, and respiratory rate—remained well within normal physiological limits in both the active and placebo groups. At the Day 56 endpoint, no statistically significant between-group differences were observed for any vital sign parameter (all *P* > 0.05; Table 7), and no participant demonstrated clinically relevant fluctuation. Comprehensive physical examination findings, encompassing general appearance, cardiovascular and respiratory system assessments, abdominal evaluation, and neurological screening, revealed no supplement-emergent abnormalities or concerning trends in either cohort. The absence of deviations in these core physiological indicators supports the overall safety and tolerability of the active supplementation during the support of women with low sexual desire.

#### Study withdrawals

A total of three participants discontinued the study (active: *n* = 1; placebo: *n* = 2), representing a low overall attrition rate of 4.28%. Importantly, none of these withdrawals were associated with adverse events, indicating that discontinuation was unrelated to supplementation tolerability. The single withdrawal in the active group and one withdrawal in the placebo arm were due to loss to follow-up, while the remaining placebo participant discontinued because of non-compliance with study procedures. The high retention rate and absence of safety-related dropouts support the strong acceptability and favorable tolerability profile of the active intervention in women with low sexual desire.

## DISCUSSION

The present study offers novel clinical insight into the potential role of Shatavari in sexual health, as literature evaluating its use specifically as an intervention for low libido in reproductive-age women is lacking to date. Comparatively, existing pharmacologic treatments for low sexual desire are associated with substantial safety and tolerability concerns, including risks of hypotension, syncope, nausea, dizziness, and the inconvenience of administration [5]. In contrast, Shatavari root extract demonstrated a better safety profile, with only mild, transient gastrointestinal complaints and no serious adverse events, suggesting potential advantages regarding patient compliance.

Shatavari had a positive effect on healthy sexual function. Specifically, improvement was observed in the FSFI (67.96%), FSEP-R (61.90%), and vaginal moisture on the modified Schirmer test (62.82%), together with significant reductions in sleep disturbance (24.81%) and fatigue (60.05%).

The most notable gains were in the lubrication (93.33%), orgasm (86.70%), and satisfaction (79.93%) domains of the FSFI in participants receiving Shatavari, substantially exceeding the placebo response (7.58%). These domains are interrelated: improved lubrication of the mucosal lining supports more effective orgasm. The findings are complemented by the modified Schirmer test, where vaginal dryness decreased after intervention, with 62.82% restoration of hydration versus 5.58% in the placebo group. This improvement in vaginal lubrication and hydration may reflect the demulcent property of Shatavari, which helps restore hydration of the mucosal membranes [6]; Shatavari has also been reported to increase pelvic blood flow, further aiding lubrication [10]. Improved lubrication in turn supports orgasm and satisfaction, and is also linked to improved desire (34.54%) and reduced pain (80.51%) during intercourse.

The DDSE data demonstrate that active supplementation led to consistent, time-dependent improvements in sexual activity, desire, lubrication, satisfaction, and reduction of pain, with additional gains in orgasm frequency and partner satisfaction emerging by Month 2. In contrast, the placebo group showed minimal or no improvement. These findings support the efficacy of the active intervention in supporting both individual and relational aspects of sexual function [7].

Building on the FSFI results, the FSEP-R showed a difference in the quality of sexual encounters after Shatavari supplementation (61.90%) versus placebo (4.44%). The event-based structure of the FSEP-R supports detection of improvements even in women whose frequency of sexual activity did not substantially change, reinforcing the scale’s ability to identify supplementation-related benefits independent of behavioral patterns driven by external factors. By measuring the qualitative components of sexual encounters-like orgasm, desire, satisfaction, etc.- this scale provides a more accurate representation of supplementation benefit.

The FSEP-R therefore offers an essential complement to global sexual function and distress measures, strengthening the multidimensional assessment of treatment effects in clinical trials.

Sleep disturbance and fatigue are important secondary factors that contribute to low libido in younger women, where these interrelated conditions play a significant role in sexual relationships and overall wellbeing. To capture this multidimensional construct, the PROMIS SD SF-8b and the FSS, well-validated patient-reported outcome measures that quantify the intensity, persistence, and functional burden of fatigue, were selected for the study. Across the intervention period, Shatavari supplementation showed significant, progressive reductions in PROMIS SD SF-8b scores relative to placebo, supporting meaningful improvements in overall sleep disturbance. Improvements were evident by Day 28 (16.76%) and strengthened further by Day 56 (21.83%), whereas placebo-treated participants exhibited minimal or no change (Day 28: −0.37%, Day 56: −0.06%). Improvements encompassed key domains of sleep quality, including satisfaction with sleep, perceived refreshment, and reduced difficulty initiating and maintaining sleep, indicating broad normalization of sleep continuity rather than isolated symptom relief. Importantly, improvements in the active group suggested a shift toward lower PROMIS sleep disturbance T-score categories. Prior validation work has established that an improvement of approximately 8 points on the PROMIS SD SF-8b represents a clinically meaningful within-patient change, suggesting that the observed reductions extend beyond statistical significance to real-world clinical relevance [23]. Improvements in disturbed sleep quality are likely to exert downstream benefits on daytime vitality, mood stability, and sexual readiness, highlighting the bidirectional relationship between sleep and sexual wellbeing [13]. Such integrative effects are particularly relevant in low sexual desire populations, where disrupted sleep often amplifies fatigue-related barriers to sexual interest and responsiveness.

The next interrelated condition is fatigue. To our knowledge, this is the first study to apply the FSS specifically within a low-libido cohort, demonstrating its validity, reliability, and sensitivity to treatment-related changes. Data from the study period indicate that participants in both groups exhibited progressive declines in FSS scores, demonstrating a meaningful reduction in fatigue burden. Improvements were significant at each follow-up visit compared with baseline, and active supplementation consistently produced greater reductions in fatigue burden (60.05%) and interference than placebo (4.45%). These findings underscore the significance of fatigue in low libido, where diminished energy, impaired vitality, and cognitive fatigue can impair sexual interest and responsiveness. The improvements observed in the Shatavari group align with the reported literature [24]. By improving sleep quality, stabilizing mood, and supporting hormonal equilibrium, Shatavari may reduce both the physical and cognitive dimensions of fatigue, thereby supporting sexual readiness and desire. This integrative effect is consistent with emerging evidence that reduced fatigue may mediate improvements in sexual function, especially in women whose desire is affected by stress, exhaustion, or diminished vitality [25]. Shatavari produced significant reductions in fatigue as early as one week after starting the intervention (*P* = 0.015), and the sustained decrease in FSS scores supports a role for the formulation in restoring vitality, energy, and functional wellbeing — factors closely linked to healthy sexual desire.

The HADS-Depression subscale, which emphasizes anhedonia rather than somatic or vegetative symptoms, offers clinically meaningful insights into changes associated with sexual desire regulation. Because diminished interest and reduced capacity for pleasure are core features of low sexual interest, improvements in HADS-D scores among Shatavari participants (within a week of intervention) suggest a positive shift in affective and motivational processes that support sexual functioning. Likewise, the HADS-Anxiety subscale, structured to assess cognitive and emotional tension while minimizing confounding somatic symptoms, effectively captures reductions in worry, tension, and autonomic hyper-arousal—factors known to inhibit sexual responsiveness. Thus, changes across both HADS sub-domains reflect broader psychophysiological improvements relevant to female sexual function. HADS also demonstrated excellent participant acceptability. Consistent with historical observations that patients find the scale easy to understand and complete, no participants reported difficulty with the weekly recall format. This recall period was a deliberate design feature, minimizing reactive anxiety associated with clinic visits and allowing scores to reflect participants’ habitual emotional state rather than transient fluctuations. The absence of HADS score inflation among physically ill participants in prior validations, paired with stable laboratory and vital sign parameters in this trial, reinforces that the psychological improvements observed were not artifacts of symptom overlap but reflect genuine mood improvement attributable to supplementation. Shatavari supplementation produced coordinated modulation of key hormonal pathways associated with female sexual desire. Collectively, the HADS data indicate that Shatavari supports improvements in sexual function alongside reductions in the everyday psychosocial burden of mild anxiety and low mood, a contributing factor to low sexual desire. These findings highlight the multidimensional impact of Shatavari on female sexual health, consistent with effects through both psychosexual and emotional regulatory pathways.

Estradiol (34.68%) and free testosterone (10.06%) showed meaningful improvements, and the increase in DHEA-S (14.99%) further indicates greater vitality and mood stability. These hormonal changes support women with low sexual desire, consistent with the reported literature underlying female sexual function [26]. The improvement observed in the active group may be due to the phytoestrogenic properties of steroidal saponins in Shatavari, which help modulate hormonal homeostasis [27].

Overall, the data from the study indicate the benefit of Shatavari in supporting healthy sexual function in women experiencing reductions in sexual desire, in addition to reducing sleep disturbances and fatigue burden from an early period after intake. Shatavari root extract data show a strong safety profile throughout the study period, with no serious adverse effects and stable vital parameters.

### Supplement implications and treatment integration

The comprehensive profile of benefits and safety demonstrated by Shatavari root extract positions it as a non-hormonal botanical supplement option for women with low sexual desire, particularly those seeking natural alternatives or those who prefer to avoid or are contraindicated for hormonal therapies. Its multidimensional effects on sexual desire, fatigue, stress, and mood make it suitable for women presenting with complex or overlapping everyday challenges to sexual wellbeing. The observed supplementation trajectory—showing early improvements within 4 weeks and maximal benefit by 8 weeks provides clear guidance for setting patient expectations regarding supplementation onset and progression.

Optimal use of Shatavari root extract as a botanical supplement includes: (1) comprehensive baseline assessment of sexual function using validated tools such as FSFI, FSEP-R, and sexual distress measures; (2) transparent communication of expected timelines for improvement; (3) follow-up assessments every 4–6 weeks to monitor supplementation response, adherence, and emerging factors influencing sexual desire; (4) incorporation of supportive behavioral interventions such as sex therapy, stress-management approaches, relationship counseling, and sleep optimization for synergistic benefit; and (5) consideration of multimodal strategies for women with complex or treatment-resistant low sexual desires. The improvements observed in everyday mood, energy, and stress indicate that Shatavari may also address psychological contributors to low sexual function, offering broader benefit across emotional and relational dimensions of sexual health.

Despite these encouraging findings, the trial had several limitations. Recruitment was challenging due to cultural sensitivities, stigma surrounding sexual health discussions, and reluctance to disclose sexual concerns, particularly among Indian women. Furthermore, sexual function is bidirectional and influenced by partner-related factors; however, sexual health information from partners was not gathered, limiting interpretation of dyadic influences. The study population may also constitute a heterogeneous group of women with varying sexual vitality phenotypes, as isolating individuals with pure hypoactive sexual desire disorder was not always feasible. Menstrual cycle variations, which can influence libido, were not systematically controlled. Additionally, the study was powered to detect changes in total FSFI scores but may have been underpowered to detect domain-specific effects, including those observed for desire and satisfaction.

Finally, as a modestly sized clinical study, the findings should be interpreted cautiously. Larger, multicentric, and longer-duration studies are needed to verify the specific benefits of Shatavari in improving desire, satisfaction, and related psychosexual parameters in women with low libido. Regardless of cultural or geographic background, chronic stress, mental load, and physical exhaustion impair female sexual wellness by dysregulating the HPA axis. Although current clinical data on Shatavari for female libido are limited to Indian populations, the adaptogenic and hormonal mechanisms demonstrated may be generalizable more broadly. These preliminary results warrant further investigation in rigorously powered studies.

## CONCLUSION

This randomized, double-blind, placebo-controlled study provides evidence that Shatavari root extract supports sexual wellbeing and related parameters in women with lifestyle-related reductions in sexual desire. Total FSFI improved significantly more with active than placebo, and the active group showed superior gains at Days 28 and 56 in the domains most relevant to low sexual desire — desire and satisfaction — suggesting domain-specific benefits not fully captured by the global composite score. These gains were reinforced at the event level on the FSEP-R, where the active group consistently outperformed placebo in arousal quality, orgasmic response, and encounter satisfaction. In conclusion, Shatavari was well tolerated and may be considered a botanical supplement that supports healthy sexual function and associated well-being in women with lifestyle-related reductions in sexual desire.

## Data Availability

De-identified participant data, study protocol, and Statistical Analysis Plan (SAP) are available upon reasonable request from the corresponding author or study sponsor. All trial data and related documents are securely maintained at Biofact Research Pvt. Ltd., Hyderabad, India, in compliance with Good Clinical Practice (GCP) and institutional data-sharing policies

## Personal thanks

The authors acknowledge the contributions of all study participants, research staff at both study sites, and the sponsor, Waleria Healthtech Pvt. Ltd.
